# Tetramethylpyrazine (TMP) protects rats against acute pancreatitis through NF-κB pathway

**DOI:** 10.1080/21655979.2019.1613103

**Published:** 2019-04-29

**Authors:** Longying Chen, Yongjun Chen, Hao Yun, Zhang Jianli

**Affiliations:** a Department of Internal medicine intensive care, the central hospital of Linyi, Yishui, Shandong, China; b Department of Traditional Chinese medicine, the affiliated hospital of Qingdao University, Shandong, China; c Department of General Surgery, The Affiliated Hospital of Qingdao University, Shandong, China

**Keywords:** Tetramethylpyrazine, nuclear factor-kappa B, apoptosis, cytokines, acute pancreatitis

## Abstract

Acute pancreatitis (AP) is a digestive disease characterized by pancreatic inflammation. *Tetramethylpyrazine* (TMP) has been effectively used to ameliorate the damage on intestinal mucosa injury in rats with acute necrotizing pancreatitis (ANP). We aim to study the protective effect of TMP on caerulein-induced AP and to explore the possible mechanism. The mice randomized into control and different experimental groups. AP was induced in mice by 6-hourly intraperitoneal (i.p) injections of caerulein (50 μg/kg at 1 h interval). TMP (i.p, 10 mg/kg, 1 h interval) was administered 3 h before caerulein injection. Administration of TMP attenuated the severity of AP as shown by the histopathology, reduced serum amylase activity and pro-inflammatory cytokines TNF-α and IL-6. Further, TMP enhances the beneficial effect by reducing caerulein-induced NF-κB activation and inducing cell apoptosis in pancreas. Therefore, inhibition of nuclear factor-kappa B(NF-κB) signals by TMP represents a potential therapeutic strategy for the treatment of acute pancreatitis.

## Graphical Abstract

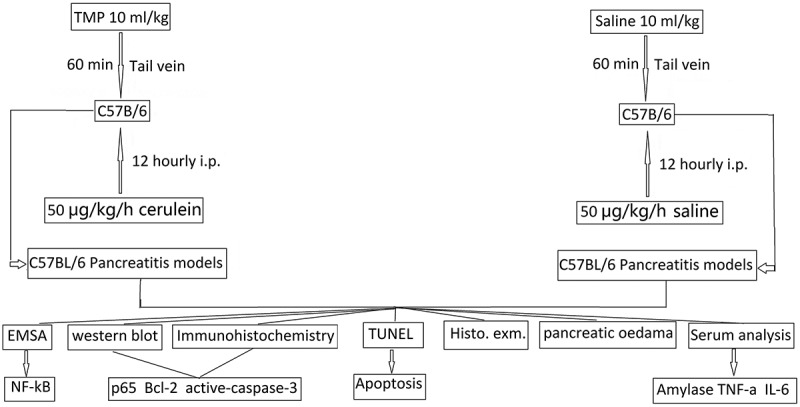

## Introduction

Acute pancreatitis (AP) is caused by toxins that induce acinar cell calcium overload, zymogen activation, cytokine release and cell death [1]. Over the last two decades, our understanding of pathogenesis has advanced, but there is still no specific therapy despite many randomized trials [2]. Specific therapy for AP is lacking and deciphering the molecular mechanisms underlying its pathogenesis will likely aid in therapeutic intervention. The transcription factor NF-κB was as a nuclear factor that binds to the enhancer element of the immunoglobulin kappa light-chain of activated B cells (thereby coining the abbreviation NF-κB) and plays a variety of evolutionarily conserved roles in the immune system. The NF-κB is activated early in acinar cells during acute pancreatitis and increases expression of multiple proinflammatory genes, increasing vascular permeability and inducing thrombosis and hemorrhage, ultimately leading to tissue necrosis [3,4]. Therefore, reducing NF-κB activation and proinflammatory mediators might be a good therapeutic strategy to attenuate AP.

Apoptosis and necrosis are two major forms of acinar cell death in AP and associated with specific morphological and biochemical features [5,6]. Pancreatic acinar cell death occurs principally via apoptosis or necrosis. Although the consequences of apoptosis and necrosis are distinct in pancreatitis, the mechanisms underlying these two types of cell death are interrelated and they both involve mitochondria [5,7]. Apoptosis, by contrast to necrosis, does not elicit an inflammatory response and hence does not injure the surrounding cells, having an active protective effect [7–9], suggesting that enhancing apoptosis could reduce the severity of AP [7,10]. A large body of evidence has demonstrated a protective role of NF-κB in most tissues and cell types. For example, targeted deletion of *p65* in mice leads to increased apoptosis in several tissues [11]. The protection by NF-κB is due to transcriptional activation of a number of antiapoptotic proteins, such as Bcl-2 and Bcl-XL [12,13].Therefore, targeting NF-κB signaling pathway could result in improved prognoses through increased apoptosis in AP.

*Tetramethylpyrazine* (TMP) is one of the major active constituents of the traditional Chinese herbal medicine, *Ligusticum wallichii Franchat* (*chuanxiong*). TMP has angiogenesis and vessel protection effect as well as anti-inflammatory function. It has been used to treat cardiovascular and inflammatory diseases clinically in China for a long time [14]. It has recently found that TMP can ameliorate the damage to pancreas and intestine caused by AP [15,16]. However, the exact mechanism underlying the effect of TMP in AP has not been completely addressed. Multiple studies have shown that TMP inhibits NF-kB activity, thereby causing inhibition of inflammatory factor release and protecting cells from damage [17–19]. In addition, TMP significantly induced apoptosis and G0/G1 arrest in osteosarcoma cells by downregulating the protein expression of nuclear NF-κB p65(P65), bcl-2 and cyclin D1 [20].

In the present study, we evaluate the therapeutic effectiveness and mechanisms of TMP on AP in rat models. Our findings show that TMP promotes apoptosis and inhibits necrosis, decreases the pancreatic inflammatory response and ameliorates acute pancreatitis in mice through inhibition of NF-κB activation, nuclear p65 translocation and bcl-2 expression.

## Materials and methods

### Ethics statement

The experimental protocol was approved by the institutional animal ethics committee of the central hospital of Linyi, with animal care performed strictly according to the guide for the care and use of laboratory animals published by the US National Institutes of Health (NIH Publication No.85–23, revised 1996). All surgery was performed under pentobarbital anesthesia, and all efforts were made to minimize suffering.

### Pancreatitis models

All animal experiments were approved by the Animal Ethics Committee of Linyi central hospital and carried out in accordance with the *International Guiding Principles for Animal Research*. For induction of pancreatitis, age – and sex-matched C57BL/6 were fasted for 18 h but provided with water and libitum (10 animals per experimental group). Mice received 6-hourly i.p. injections of saline (control) or 50 μg/kg/h cerulein (Sigma-Aldrich, St. Louis, MO, USA) in saline. Animals were killed by CO_2_ asphyxiation 1 h after the final caerulein injection. The blood and tissue samples were collected for studies. To assess the severity of cerulein-induced pancreatitis, H&E-stained tissue sections from all experimental groups were graded by two blinded independent observers, using the method described previously [22].

### TMP administration

TMP (purity >98%) was purchased from Sigma. The dosages of TMP were chosen based on previous reports [23,24]. TMP was administered *via i.p* in a volume of 10 ml/kg for 1 h starting 3 h before the administration of cerulein for induction of pancreatitis and control animals were given i.p injection of saline for 1 h.

### Electrophoretic mobility shift assay (EMSA)

Electrophoresis Mobility Shift Assay (EMSA) was performed as reported previously [25]. Briefly, nuclear and cytoplasmic extraction reagents were used to extract the nuclear proteins of tissues. The BCA method was used to measure protein concentration. The NF-κB probe *AGT TGA GGG GAC TTT CCC AGG C* (Santa Cruz Biotechnology, Shanghai, China) was labeled with [α-^32^P] dCTP, which were incubated with 10 µg nuclear extracts for 30 min at room temperature. Anti-p65 antibody (BD Pharmingen) was used to observe a supershift. The reaction mixture was electrophoresed on 4% polyacrylamide gels, and the gel with separated samples was dried and subjected to autoradiography using phosphor screens at −80°C.

### Western blotting

Pancreas tissues were lysed in 20 mM Tris-HCl, pH 8.0, 100 mM NaCl, 1 mM EDTA, 0.5% Nonidet P-40, 5 mM NaF, 50 mM 2-glycerophosphate, and protease inhibitors (Roche, Shanghai, China). Then, the lysates above were centrifuged at 12,000 rpm at 4°C for 10 min. Samples were separated through an SDS-PAGE, transferred to Immobilon P membranes, and western blotting was performed with specific antibodies against p65 (Santa Cruz Biotechnology), cleaved-caspase-3, bcl-2 and, as a loading control, an anti-GAPDH antibody (Sigma). Appropriate fluorescent dye-labeled secondary antibodies were used to allow detection with the Odyssey Infrared Imaging System (LI-COR Biosciences) as previously described [26]. Blots are representative of at least three experiments.

### Immunohistochemistry (IHC)

Immunohistochemistry was performed in accordance with the instructions of the SP-9001 Kit (Beijing Nobleryder Technology Co. Ltd., Beijing, China). The paraffin-embedded pancreatic tissue blocks obtained from the mice of the normal and AP groups were placed at room temperature for 30 min. The tissues were then fixed with acetone at 4°C for 10 min, dewaxed, rehydrated, exhaust the endogenous peroxidase activity, incubated with rabbit anti-NF-Kbp65,bcl-2 antibody (Abcam Inc.,Cambridge, MA, USA) at 4°C overnight, then incubated with a corresponding biotinylated goat anti-rabbit IgG secondary antibody as the previously described[27]. The samples were dehydrated with graded ethanol, permeabilized with xylene and mounted by neutral balsam. Phosphate-buffered saline (PBS) was regarded as the control during the replacement of the primary antibody. The experiment was repeated 3 times. The scores of staining intensity and cell rate of positive expression were calculated using the OlymPusDp70 image acquisition analyzer. The scale of staining intensity was as follows: 0, no staining; 1, weak staining; 2, moderate staining; 3, strong staining. The criteria for the cell rate of positive expression were as follows: 0, <1%; 1, <10%; 2, <50%; 3, <80%; 4, >80%; The final score was calculated based on staining intensity and cell rate of positive expression: 0–2, negative (-); 3–5, positive (+); 6–7, strongly positive (++).

### TUNEL assay

For detection of cell death, the TUNEL (**T**erminal deoxynucleotidyl-transferase mediated d**U**TP **N**ick **E**nd **L**abelling) method was performed as previously described [28] and according to the Apop-Tag Plus kit (Chemicon Internacional, Shanghai,China). Sections adhered to silanized slides (3-aminopropyltrithoxysylane – Sigma-Aldrich Chemical Co., St. Louis, USA) were treated with 20 μg/ml proteinase K (Sigma- Aldrich Chemical Co., St. Louis, USA) and immersed in 3% hydrogen peroxide. After immersion in equilibration buffer for 20 min, the sections were incubated in TdT enzyme (Terminal deoxynucleotidyl Transferase) at 37°C for 1 h in a humidified chamber. The reaction was stopped by immersion in a stop/wash buffer for 20 min and incubated in anti-digoxigenin-peroxidase in a humidified chamber at 37°C for 30 min. The reaction was revealed with 0.06% 3.3‘-diaminobenzidine tetrahydrochloride (DAB-Sigma-Aldrich Chemical Co., Hangzhou, China) and counterstained with Carazzi’s hematoxylin. Sections of involuting mammary gland, provided by the manufacturer of the Kit, were used as positive controls for the TUNEL method. The sections used as negative controls were submitted to the same protocol, except the step of incubation in the TdT enzyme. Total apoptotic cells were calculated by counting the number of TUNEL-positive cells.

### Histological examination

For light microscopy, fresh specimens of murine pancreas were fixed in 4% paraformaldehyde in PBS (pH 7.4). The tissues were embedded in paraffin, and 5 mm sections were processed for hematoxylin and eosin (H&E) staining by standard procedures. Multiple randomly chosen microscopic fields from at least three mice in each group were examined and scored by two pathologists in a blind manner based on the presence of vacuolization, interstitial edema, interstitial inflammation, the number of acinar cell necroses, as previously described [29]. The scoring assessment was performed on a scale of 0–3 (0 being normal and 3 being severe) on each parameter mentioned above and the sum of the scores were used to evaluate the severity of acute pancreatitis.

### Quantification of pancreatic oedema

Pancreatic edema was estimated as water content. After exsanguination of the rat, a portion of the pancreas about 1 g in wet weight was excised and weighed. The tissue was dried in a vacuum centrifuge for 36 h at 60°C and reweighed. The resulting dry weights were subtracted from the original wet weights, yielding an estimate of the water content of the pancreata. The difference between wet weight and dry weight was calculated. The increased water content of the tissue was expressed as a percentage of the water content of a normal rat pancreas.

### Serum amylase analysis

The extent of the pancreatic injury was determined by measuring serum amylase level using Phadebas reagent (Magle Life Sciences) as recommended by the supplier.

### Measurement of TNF-α and IL-6

The serum levels of TNF-α and IL-6 were determined using enzyme-linked immunosorbent assay (ELISA) kits (EIAab, Shanghai, China) as the manufacturer's instruction.

### Statistical analysis

Results were expressed as mean ± standard error of the mean from multiple separate experiments. Statistical analysis was performed using *t-*tests with SPSS version 16 software (IBM, Portsmouth, UK). Statistical significance was set at *P* values <0.05.

## Results

### TMP protected from caerulein-induced pancreatitis in mice

**Figure 1.**
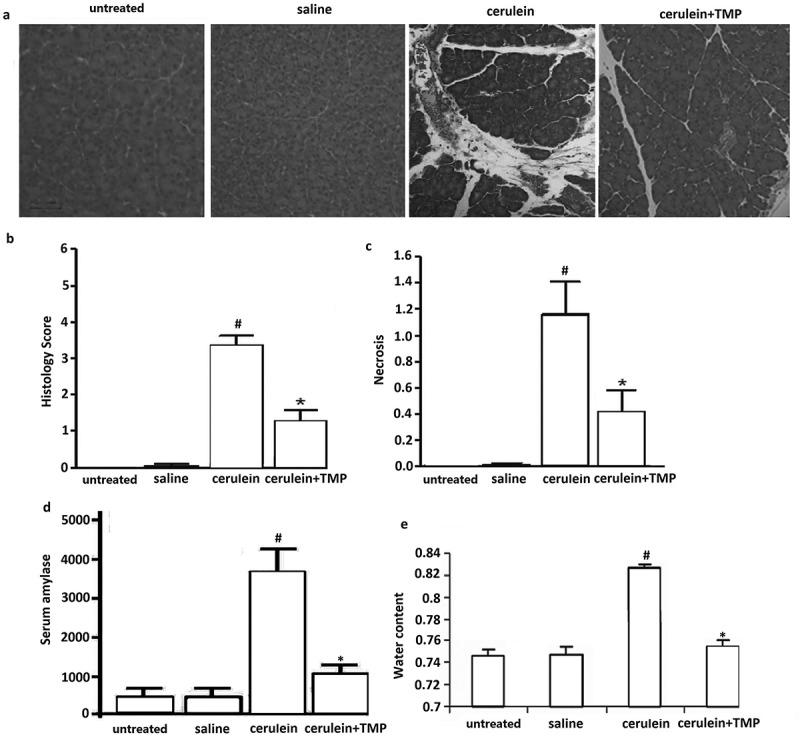
Evaluation of cerulein-treated and TMP-treated pancreatic tissue damage in mice. (a), Pathological morphology of pancreatic tissues in each group measured by haematoxylin-eosin (HE) staining. (b), Representative H&E images of pancreas histology slides from treatment groups; (c), Necrotic cells were counted on H&E-stained pancreas sections as percentage of total cells. D, Effect of TMP on serum amylase activity (U/L) of cerulien-induced acute pancreatitis in mice (i.p.). (e), Effect of TMP on pancreatic water content in the cerulein treated mice. vs untreated and saline,^#^*p*< 0.05; vs cerulein,*p< 0.05.

Mice were sacrificed 1 h after the final injection of caerulein. From inspection of H&E-stained sections, it was clear that acute pancreatic damage had been induced by the caerulein in pancreata of mice. Caerulein-injection led to a pronounced pancreatic edema, hemorrhage, leucocyte infiltration and necrosis (Figure1(a)). However, each of these features was markedly reduced by the prior administration of TMP (Figure1(a)). Furthermore, administration of TMP significantly decreased histological scoring and necrosis compared with the cerulein alone treated mice (Figure1(b,c)).

The evidence of pancreatic injury induced by caerulein is generally confirmed by an increase in plasma amylase. Thus, the effect of TMP treatment on plasma amylase in AP was evaluated, especially in comparison with the corresponding values in saline pretreated mice. As shown in Figure1(d), mice treated with caerulein injections, pancreatitis was manifested by a significant rise in serum amylase activity compared to the mice injected with hourly saline only (*P*< 0.05). However, among the TMP treatment groups, serum amylase was significantly reduced in mice (*P*< 0.05).

To determine the percentage of pancreatic water in the various groups, tissues were dried for 36 h at 90°C. The results showed that caerulein injections alone significantly increased relative water content in the pancreata. In contrast, the TMP treated pancreata exhibited only slight increases in the relative water content following caerulein injections (Figure 1(e), p < 0.05).

### DHA reduced serum TNF-α and IL-6 in the cerulein-induced mice

**Figure 2.**
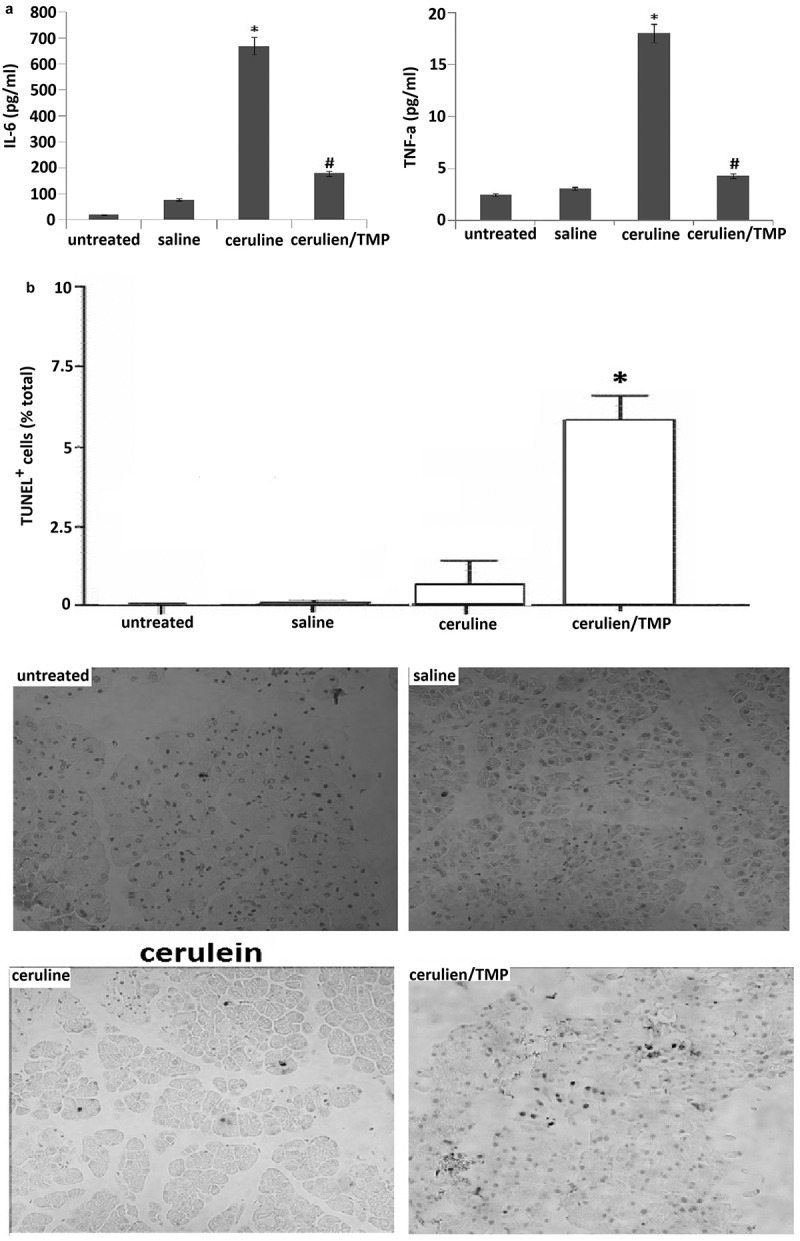
TMP inhibits serum TNF-α and IL-6 levels and promotes apoptosis in cerulein-induced pancreatitis. (a), Serum levels of interleukin (IL)-6 and tumor necrosis factor (TNF)-αwere detected 12 h after induction of acute pancreatitis or treated with TMP before the induction of acute pancreatitis. (b), Cell apoptosis was detected by TUNEL assay. Data are presented as mean ± SEM.*P< 0.05; ^#^*P*< 0.05.

We examined serum IL-6 and TNF-αlevels, and the results showed a significantly enhanced level of IL-6 and TNF-α in the cerulein-induced pancreatitic models, and this was significantly reduced in TMP-treated mice (Figure 2(a)).These data demonstrate that TMP inhibits serum TNF-α and IL-6 levels in cerulein-induced AP in mice.

### TMP induces apoptosis in cerulein-induced pancreatitis

We have analyzed apoptosis using TUNEL assay on pancreatic tissue sections (Figure 2(b)). Quantification for these assay data confirmed that there were more apoptotic cells in the pancreatic tissue of TMP-treated mice (5.6 ± 1.2) than that of in the cerulein alone treated mice (1.2 ± 0.3) (*P*< 0.05). These results demonstrate the TMP treatment induces an apoptotic response in pancreatic tissue.

### TMP inhibited cerulein-induced activation of NF-kB in the pancreas

**Figure 3.**
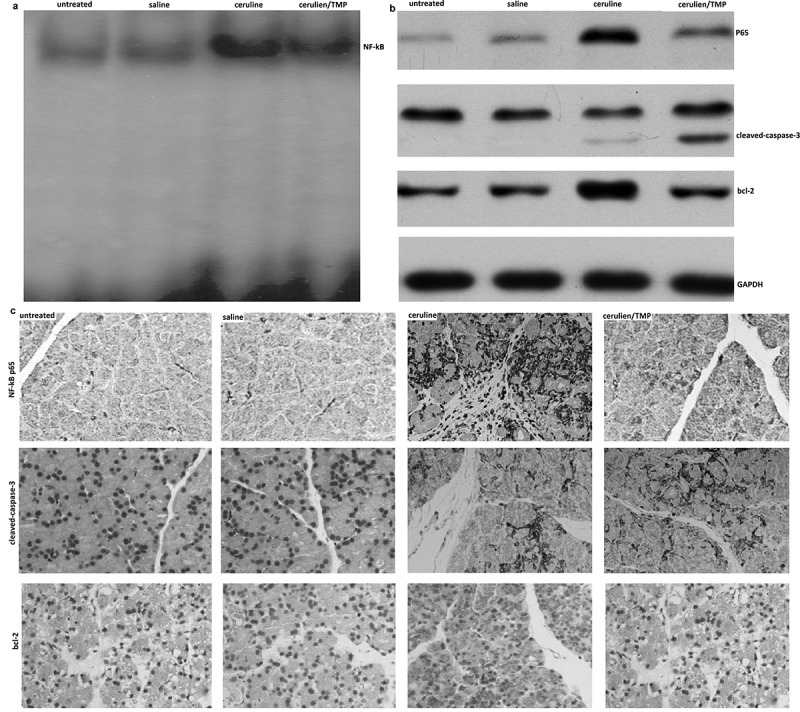
Changes in pancreatic levels of NF-Kb P65 nuclear transcription, Bcl-2 protein expression and caspase-3 activation in models of acute pancreatitis. (a), EMSA assay; (b), western blot assay; (c), Immunohistochemistry (IHC).

Here, we investigated the effect of TMP on cerulein-induced activation of NF-κB by determining the NF-κB-DNA binding activity in the pancreas. As shown in Figure 3(a), NF-κB was activated in the pancreas of cerulein-induced acute pancreatitis. However, TMP suppressed the cerulein-induced activation of NF-κB in the pancreas. In addition, a significant increase in nuclear transcription of p65 in the pancreas of cerulein-induced acute pancreatitis by Western blot assay (Figure 3(b)) and immunohistochemistry assay (Figure 3(c)). However, nuclear transcription of p65 was significantly inhibited in TMP-treated pancreas (Figure 3(a–c)).

Western blot analysis showed that the prosurvival proteins Bcl-2 were present in normal rat pancreas, and were up-regulated in models of acute pancreatitis (Figure 3(b-c)). Up-regulation of pancreatic Bcl-2 protein was detected in all models examined, namely pancreatitis induced by cerulein in mice. However, the levels of bcl-2 were inhibited by TMP (Figure 3(b-c)). In addition, caspase-3 was activated in TMP treated pancreas, indicating that TMP increased cell apoptosis in mice pancreas (Figure3 (b-c)).

## Discussion

The current therapeutic guidelines for acute pancreatitis (AP) include the following: intravenous fluid replacement, dietary changes, analgesics, inhibitors of pancreatic secretion (somatostatin and its analog, octreotide), L-arginine, calcium ion antagonists, and different inflammatory mediator inhibitors [30–33]. Unfortunately, the use of standard drugs in acute pancreatitis is still disappointing.

Chinese herbal formulas have upheld the holistic therapeutic philosophy for thousands of years, which consists of two or more appropriate medicinal plants or animals according to the prescription compatibility principle of traditional Chinese medicine (TCM) formulations and determining the dosage and usage of each medicine [34]. The components of Chinese herbal formulas are complex and diverse, and the main treatment mechanism seems reasonable. These herbal formulas are an organic combination of many effective components, which have a multi-target effect on the disease in the body by multiple pathways [35]. An increasing number of Chinese herbal formulas have been reported to have significant anti-AP effects, and have become a treatment option in many hospitals for AP [36–38].

Pharmacologic studies have demonstrated that *Tetramethylpyrazine* (TMP, also known as *Ligustrazine*), an intravenous drug made from traditional Chinese herbs, is able to reduce inflammatory responses [39] and activation of apoptosis [40]. It is capable of attenuating the severity of AP and protecting multiple organ injury in rats with AP [41]. Our results demonstrate that cerulein-induced AP shows a more acute pancreatitis model with deteriorated pancreatic inflammation, increased serum cytokine level, evident local acinar necrosis, as well as drastic systemic inflammatory responses. Importantly, all parameters quantifying the severity of AP were reduced, but cell apoptosis was increased in TMP-treated AP as compared with the cerulein-induced AP.

Many factors are involved in the process of the pathogenesis of AP, and the accurate mechanisms are still unclear. Evidences suggest that proinflammatory cytokines, such as IL-1β, TNF-α, and IL-6, act as mediators of acute pancreatitis. IL-6 is a proinflammatory cytokine associated with acute phase responses during inflammation, and elevated levels of IL-6 have been observed in patients with AP and are determinants of disease severity [42]. TNF-α is produced in pancreatic acinar cells in experimental AP model. It is an activator of immune cells and regulates the synthesis of other pro-inflammatory cytokines [43]. It is critical for the therapy of AP by reducing the cascade of cytokines at the early stages and ameliorating the disease and its systemic complications. The cerulein-induced pancreatitis was a sudden inflammation in the pancreas with extensive infiltration of leukocytes and excessive production of amylase. Dang et al. have reported that TMP can ameliorate microcirculatory disorder and alleviate the damage to the pancreas and stomach [44]. Zhang et al. have reported that TMP can ameliorate the condition of microcirculatory disorder and the damage of pancreas and kidney [45]. In this study, TMP greatly reduced the infiltration of leukocytes and pancreas damage and decreased the IL-6 and TNF-*α* level in AP models by inhibiting the activation of NF-*κ*B. Inhibition of NF-*κ*B has been shown to improve survival rates in rats with taurocholate-induced pancreatitis [46]. Therefore, treatment with TMP could be an efficacious and promising remedy in the treatment for AP.

Apoptosis in pancreatic acinar cells is mediated mainly by activation of caspases. Of importance, it has been increasingly recognized that caspases not only mediate apoptosis but also protect from necrosis and decrease the severity of pancreatitis [45]. NF-κB-dependent anti-apoptotic gene transcriptional activation and caspase system inhibition has been demonstrated to be crucial in regulating cell death in pancreatitis [6,47]. Chen et al. have reported that TMP alleviates acute pancreatitis by accelerating acinar cell apoptosis at early phase via the suppression of p38 and Erk MAPK pathways [48]. Our findings indicated that TMP reduced the activation of NF-kB and increased the subsequent cleaved-caspase-3 expression and reduced the subsequent bcl-2 expression, which resulted in increased apoptosis and reduced severity of cerulein-induced AP.

In conclusion, our study demonstrates that TMP could protect against AP by reducing the activation of NF-*κ*B,blocking inflammatory factors and increasing cell apoptosis, thereby collectively reduced the severity of AP. Thus, TMP represents a potential therapeutic agent in the management of AP.

## Highlights

1. Tetramethylpyrazine (TMP) effectively reduced the cerulein-induced pancreatic histological damage.
2. Tetramethylpyrazine inhibits amylase activity and pro-inflammatory cytokines TNF-α and IL-6.

Tetramethylpyrazine blocks NF-κB activation and inhibits of pro-apoptotic signals in AP models.
